# When alternative becomes essential: The role of mitochondrial glycerol-3-phosphate dehydrogenase

**DOI:** 10.1073/pnas.2535701123

**Published:** 2026-02-25

**Authors:** Léa Herpe, Mélanie Aminot, Nicolas Pichaud

**Affiliations:** ^a^New Brunswick Centre for Precision Medicine, Moncton, NB E1C8X3, Canada; ^b^Department of Chemistry and Biochemistry, Université de Moncton, Moncton, NB E1A 3E9, Canada

**Keywords:** mitochondria, glycerol-3-phosphate, *Drosophila*, reactive oxygen species, comparative physiology

## Abstract

Complex I is known as the primary entry point for electrons within the mitochondrial electron transport system (ETS). However, the glycerol-3-phosphate (G3P) shuttle, composed of cytosolic and mitochondrial G3P dehydrogenase (cG3PDH and mtG3PDH, respectively), transfer reducing equivalents from the cytosol to the mitochondrial matrix. The mtG3PDH feeds electrons into the ETS via FADH_2_ oxidation, but with theoretically lower energy conversion efficiency than complex I. It is thus believed to be an “alternative” pathway, only supporting mitochondrial respiration when complex I fails. mtG3PDH also plays an important role in reactive oxygen species (ROS) production. To investigate the role of this understudied protein in mitochondrial bioenergetics and redox homeostasis, we generated *Drosophila melanogaster* mutant lines for mtG3PDH (GPO1) using a CRISPR/Cas9-based approach and determined several physiological and metabolic parameters. A drastically higher mortality rate was observed among the GPO1 flies, as well as a lethargic behavior characterized by an inability to climb. These results are in accordance with an impaired mitochondrial efficiency (ATP/O) mainly due to decreased ATP production (~60% decrease) and O_2_ consumption (~33% decrease), rather than elevated ROS. In fact, GPO1 flies produced ~70% less ROS than controls, likely due to the reduced direct and reverse electron transfer-related ROS production from mtG3PDH. These results support an essential role of mtG3PDH in mitochondrial bioenergetic, challenging its alternative aspect, and confirming its importance in mitochondrial redox homeostasis.

Mitochondria produce ATP via the oxidative phosphorylation process (OXPHOS) driven by the electron transport system (ETS). Electron entry into the ETS occurs through multiple complexes that oxidize either NADH-linked substrates (complex I–CI) or FADH_2_-linked substrates (complex II–CII, mitochondrial glycerol-3-phosphate dehydrogenase–mtG3PDH, electron transferring flavoprotein, among others), whose availability varies with substrate supply and cellular or environmental signals, a process known as mitochondrial flexibility (1). CI is widely regarded as the main electron entry point, coupling mitochondrial NADH oxidation to proton pumping and supporting most ATP synthesis (2). In contrast, FADH_2_ alternative pathways do not pump protons directly, suggesting that they may serve other metabolic or regulatory purposes.

Among those, the glycerol-3-phosphate (G3P) shuttle (Fig. 1*A*) converts cytosolic NADH to mitochondrial FADH_2_ via two enzymes: i) cytosolic G3P dehydrogenase (cG3PDH), which converts dihydroxyacetone phosphate (DHAP), from glycolysis, into G3P using cytosolic NADH; and ii) mtG3PDH, located on the outer surface of the inner mitochondrial membrane (3), which oxidizes G3P back to DHAP, transferring electrons to the ETS ubiquinone (Q) via oxidation of FADH_2_ to FAD (2). mtG3PDH is often described as an “alternative” electron entry point due to its lower ATP yield (1.5 ATP per FADH_2_) compared to NADH-linked pathways (2.5 ATP), particularly when contrasting with the malate-aspartate shuttle (4). However, emerging evidence indicates that mtG3PDH plays a critical role in supporting mitochondrial respiration when CI is impaired, particularly during conditions of mitochondrial inflexibility, such as dietary changes or elevated temperatures in insects (5, 6).

**Fig. 1.**
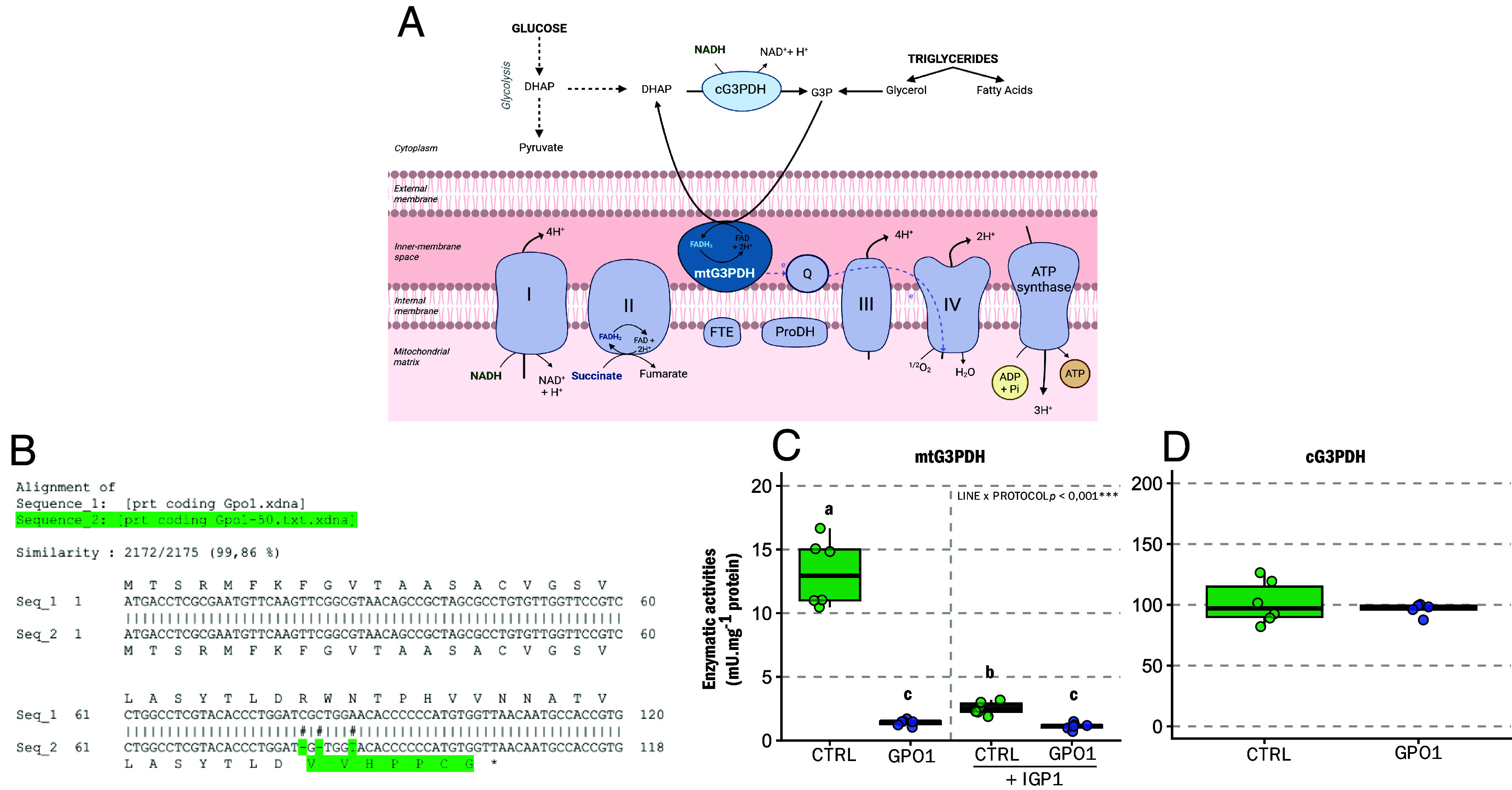
GPO1 flies genetic modification successfully reduced mtG3PDH enzymatic activity without affecting cG3PDH activity. (*A*) Schematic representation of the G3P-shuttle. Created with BioRender®. (*B*) Alignment of the Gpo1 coding sequence between control (CTRL) and CRISPR-edited (GPO1) flies (see *SI Appendix* for details). (*C*) Enzymatic activity of mtG3PDH, with or without mtG3PDH inhibitor, iGP-1. Different letters represent significant differences between lines and protocol (n = 5 to 6). (*D*) Enzymatic activity of cG3PDH (n = 5 to 6).

In addition to its role in shuttling electrons into the mitochondrion, mtG3PDH also plays an important role in the production of reactive oxygen species (ROS), thereby influencing mitochondrial redox state (7, 8). First, when mtG3PDH oxidizes FADH_2_ to FAD, it generates O_2_^•-^ on both sides of the mitochondrial inner membrane. Moreover, high mtG3PDH activity can drive reverse electron transfer (RET), by favoring hyper-reduction of ubiquinone, forcing electrons back upstream in the ETS, thus increasing ROS production at the CI_q_ site of CI (9).

Despite its seemingly important contribution to energetic and redox homeostasis, mtG3PDH is largely understudied. To further investigate the function of this complex, mutant *Drosophila melanogaster* fly lines (GPO1) were generated using CRISPR/Cas9 mutagenesis in the *Glycerol Phosphate Oxidase 1* (*Gpo1*) gene, encoding mtG3PDH (Fig. 1*B*). Specific modification was confirmed by enzymatic activity assays and sequencing (Fig. 1 *C* and *D*). Survival and climbing performance were determined in 10-day-old male flies from both control (CTRL) and GPO1 lines. Flies of the same age were also collected for mitochondrial isolation from thoraxes to measure ATP production, mitochondrial oxygen consumption, and H_2_O_2_ emission rates, under several conditions.

## Results and Discussion

**Fig. 2.**
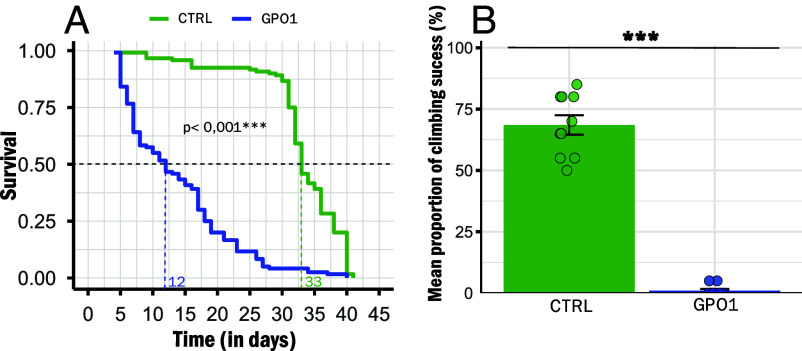
Mutation of mtG3PDH severely impairs fly survival and climbing capacities. (*A*) Survival curve: Data were analyzed by a log-rank test (n = 120). Dashed lines indicate median lifespans for each line. (*B*) Climbing capacities: Data are means ± SEM. *** represents significant differences at *P* < 0.001 between the two lines of flies (n = 10 vials).

GPO1 flies had a significant reduction in survival (log-rank χ^2^ = 170, *P* < 0.001; Fig. 2*A*), with a median lifespan of 12 d compared to 33 d in the CTRL group. GPO1 flies also exhibited severe locomotor impairment with only 1% of individuals able to climb to the top of vials within 30 s (*P* < 0.001; Fig. 2*B*), compared to 68.5% for the CTRL group.

**Fig. 3.**
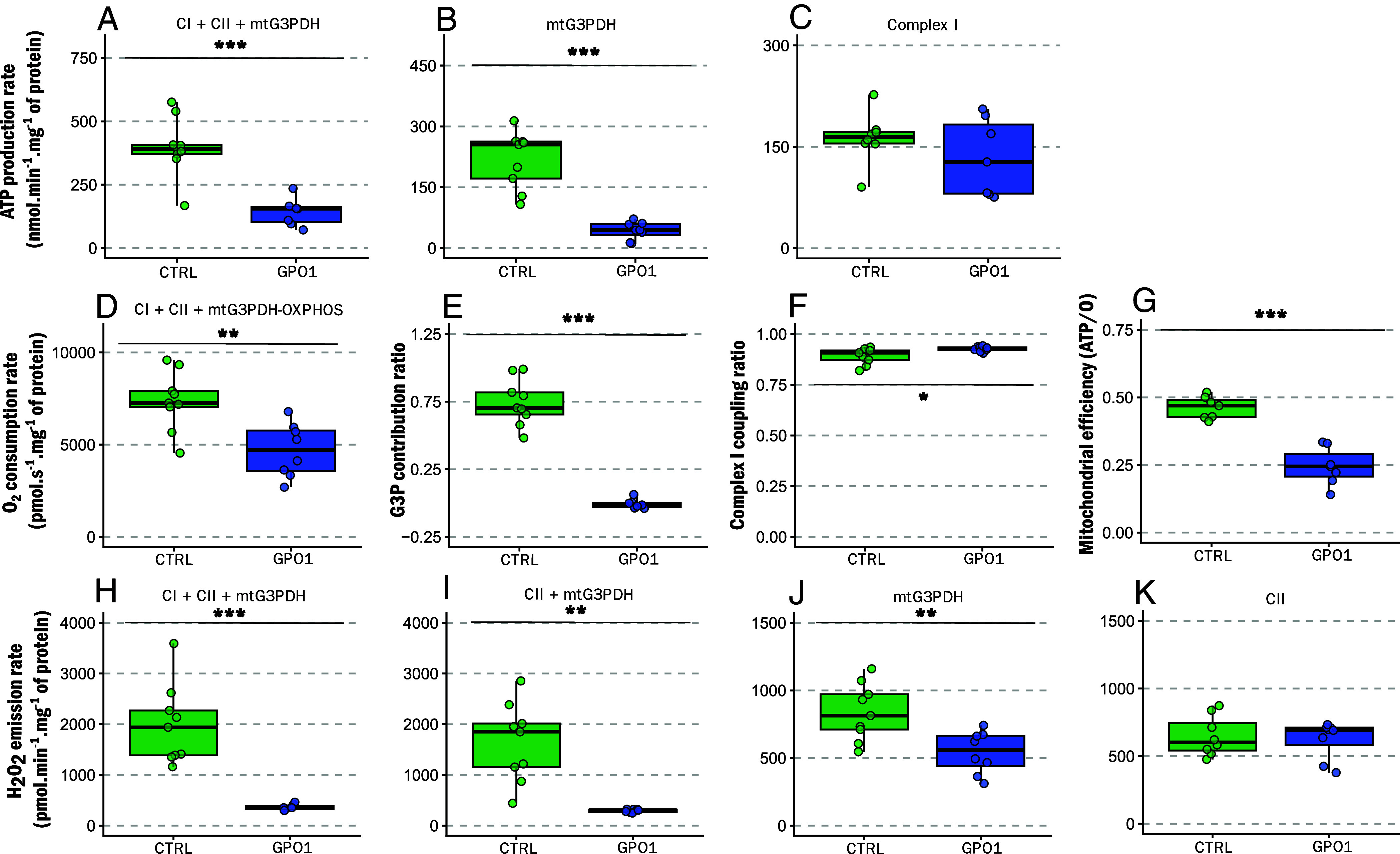
Mutation of mtG3PDH (GPO1) affected mitochondrial bioenergetic and redox state. (*A*–*C*) ATP production for (*A*) Complex I (CI), Complex II (CII), and mtG3PDH combined (CI+CII+mtG3PDH); (*B*) mtG3PDH and (*C*) CI; (*D*) Oxygen consumption rate during CI+CII+mtG3PDH-OXPHOS; (*E*) Contribution of G3P to mitochondrial O_2_ consumption; (*F*) Complex I coupling ratio; (*G*) Mitochondrial efficiency ATP/O ratio when CI, CII, and mtG3PDH are fueled and calculated from (*A* and *D*); (*H*–*K*) H_2_O_2_ emission rates measured for (*H*) CI+CII+mtG3PDH, (*I*) CII+mtG3PDH, (*J*) mtG3PDH, and (*K*) CII. Asterisks represent significant differences between the two fly lines (n = 6 to 9) (*P* < 0.01**; *P* < 0.001***).

Consistent with these phenotypes, mitochondrial functions were drastically impaired in GPO1 flies. Overall ATP production was significantly reduced (*P* < 0.001; Fig. 3*A*), primarily due to a decreased contribution of mtG3PDH (*P* < 0.001; Fig. 3*B*), while CI contribution remained unchanged (Fig. 3*C*). Similarly, oxygen consumption during CI+CII+mtG3PDH-driven OXPHOS was significantly lower in GPO1 flies (*P* < 0.01; Fig. 3*D*), once again driven by the reduced contribution of G3P to mitochondrial respiration (*P* < 0.001; Fig. 3*E*). CI coupling efficiency was only slightly affected (*P* < 0.05; Fig. 3*F*).

Taken together, ATP production and O_2_ consumption were used to calculate mitochondrial efficiency (ATP/O), when CI, CII, and mtG3PDH were fueled (i.e., results from Fig. 3 *A* and *D*), which was found to be markedly reduced in GPO1 flies (*P* < 0.001; Fig. 3*G*). This clearly indicates impaired coupling between ATP production and oxygen consumption, consistent with the severe locomotory deficit and decreased survival observed in GPO1 flies. Together, this demonstrates the essential role of mtG3PDH in maintaining mitochondrial bioenergetics and overall physiological processes.

Regarding ROS production, GPO1 flies showed a marked decrease in H_2_O_2_ emission when substrates for CI, CII, and mtG3PDH were provided (CI+CII+mtG3PDH, *P* < 0.001; Fig. 3*H*), as well as when CI was not supplied (CII+mtG3PDH, *P* < 0.01; Fig. 3*I*). This reduction was driven by a substantial decrease in the contribution of mtG3PDH to ROS production at both of its normally prominent sites: directly at the mtG3PDH site (*P* < 0.01; Fig. 3*J*) and at the CI_q_ site, which is supported under the CII+mtG3PDH condition (Fig. 3*I*). Because mtG3PDH-linked emission rate was measured in the presence of rotenone to inhibit CI–mediated reverse electron transfer, these values likely represent a conservative estimate of the full ROS-generating capacity of mtG3PDH. In contrast, CII-derived H_2_O_2_ emission did not differ between lines (Fig. 3*K*). These results show the major role of mtG3PDH in the mitochondrial redox state. Though mtG3PDH impairment may lead to less oxidative damage, it also likely impairs ROS signaling of key mitochondrial renewal pathways such as apoptosis and biogenesis (10), thus contributing to the reduced survival in GPO1 flies.

Overall, these results overturn the notion of mtG3PDH as simply an alternative pathway and instead establish it as a key determinant of mitochondrial functions and survival. These findings expand the relevance of mtG3PDH, suggesting a broader role in mitochondrial physiology and highlighting its capacity to engage multiple pathways to fulfill its function (1). Furthermore, its influence on mitochondrial bioenergetics may extend to other important cellular processes as this protein has been associated with several pathologies in humans such as neurological conditions, metabolic diseases, and cancers (11–14).

## Materials and Methods

Detailed methods and statistical analysis are described in the *SI Appendix*.

## Supplementary Material

Appendix 01 (PDF)

The SI Appendix has been updated to correct the text.

The SI Appendix has updated to correct the text.

## Data Availability

Dataset have been deposited in Mendeley data (15).
