# Supplementary material for: When alternative becomes essential: The role of mitochondrial glycerol-3-phosphate dehydrogenase
Source: Proc Natl Acad Sci U S A. 2026 Feb 25;123(9):e2535701123. doi: 10.1073/pnas.2535701123 (PMC12956892; doi:10.1073/pnas.2535701123)
Supplement: Supplementary file 1 — Appendix 01 (PDF) [file pnas.2535701123.sapp.pdf]

SUPPORTING INFORMATION

When alternative becomes essential: the role of the Mitochondrial Glycerol-3-Phosphate Dehydrogenase.

Léa Herpe[1,2], Mélanie Aminot[1,2], Nicolas Pichaud[1,2*]

[1] New Brunswick Centre for Precision Medicine, Moncton, NB, Canada, E1C8X3
[2] Department of Chemistry and Biochemistry, Université de Moncton, Moncton, NB, Canada, E1A3E9

*Corresponding author: Nicolas Pichaud

EXTENDED METHODS

Mutant line generation: Targeted mutations in the *Drosophila melanogaster Glycerol phosphate oxidase 1 (Gpo1*; CG8256) gene were generated using CRISPR/Cas9. Editing was performed by Genome ProLab (Sherbrooke, QC, Canada). A Drosophila stock expressing a single guide RNA (sgRNA) targeting the 5′ region of the *Gpo1* coding sequence (Bloomington stock 79749; sequence: CCTCGTACACCCTGGATCGCTGG) was crossed to a germline Cas9-expressing line (Bloomington stock 54591; y[1] M{w[+mC]=nanos-Cas9.P}ZH-2A w[*]). Cas9-induced double-strand breaks at the target site were repaired by non-homologous end joining (NHEJ), generating indel alleles. Edited G1 individuals were identified by PCR, and the confirmed mutant allele was balanced using the CyO chromosome to maintain a stable stock. The CyO balancer, which carries the *Cy1* gene, allowed straightforward phenotypic identification of genotypes: heterozygous flies (*Gpo1*/CyO) displayed curly wings, whereas homozygous mutants (*Gpo1/Gpo1*) had straight wings. All experiments were performed using homozygous individuals. Mutations were validated by Sanger sequencing. The resulting *Gpo1* mutant line (GPO1) carries a 2 bp deletion and 1 bp substitution, introducing a frameshift after amino acid 27 and producing a truncated protein with seven aberrant residues followed by a premature stop codon. Control flies (CTRL) underwent identical handling but retained an unmodified *Gpo1* sequence.

Fly rearing: Both CTRL and GPO1 lines were maintained at 24 ± 0.1 °C with a 12 h: 12 h light: dark cycle. Male from both lines of less than 1 day old were collected and put in vials at a density of 20 flies per vials containing a standard diet consisting of agar-agar (5 $g.L^{-1}$), sugar (6 $g.L^{-1}$), yeast (27 $g.L^{-1}$), cornmeal (53 $g.L^{-1}$) mixed in 1 L of tap water and supplemented with methyl-p-hydroxybenzoate dissolved in 95% ethanol (10% w/v) to prevent mold growth, and 0.4% (v/v) propionic acid to prevent mite contamination.

Survival: Flies were put in vials at a density of 20 flies per vial (n=120). Mortalities were recorded every 2-3 days, and food was changed after a maximum of 5 days.

Climbing test: The climbing test (negative geotaxis) was performed as previously described (1). Ten flies were transferred to empty vials (9.4 cm tall × 2.5 cm wide). Trials were then initiated by gently tapping the vials to position all flies at the bottom of the vial. The number of flies that successfully reached the top quarter of the vial within 30 sec was counted. These assays were repeated two times for each vial and performed in 2 trials across different days (n=10 vials).

mtG3PDH enzymatic activity: Activity of mtG3PDH was measured spectrophotometrically in thorax homogenates prepared in PBS (137 mM NaCl, 2.7 mM KCl, 10 mM Na_2HPO_4, 2 mM KH_2PO_4, pH 7.4), using the 2,6- dichlorophenolindophenol (DCPIP, 2 µM)-linked reduction in presence of G3P (15 mM) and malonate (5 mM). Linear changes in absorbance at 600 nm were measured at 24 °C with a BioTek Synergy H1 microplate reader (Biotek®), in presence or absence of mitochondrial GPDH inhibitor, iGP-1 (100 µMm, Sigma Aldrich) (2). Control assays without thorax homogenates were performed to validate the enzymatic assay, and the corresponding background activity was subtracted from the values obtained in the presence of homogenates and the reaction medium. Additionally, controls with homogenates but without G3P were performed to ensure the specificity of the reaction (data not shown). Protein content of homogenates were assayed using the bicinchoninic acid method (3), with bovine serum albumin (BSA) as a standard and activity is expressed as $U.mg^{-1}$ protein, where U represents 1 µmol of substrate transformed into product in 1 min.

cG3PDH enzymatic activity: Activity of cGP3DH was assessed in thoraces homogenates prepared in PBS. The rate of oxidation of NADH (0.2 mM) was recorded at 340 nm with a BioTek Synergy H1 microplate reader (Biotek®) in the presence of dihydroxyacetone phosphate (DHAP, 0.15 mM) at 24 °C in Tris-HCl buffer (50 mM of Trisma Base, 0,1% v/v Triton X-100, 1 mM $MgCl_2$, 1 $g.L^{-1}$ BSA in distilled water, pH 7.8). Activity is expressed as $U.mg^{-1}$ protein, where U represents 1 µmol of substrate transformed into product in 1 min. Control assays without thorax homogenates were performed to validate the enzymatic assay, and the corresponding background activity was subtracted from the values obtained in the presence of homogenates and the reaction medium. cG3PDH activity

was also tested in the presence of the mtG3PDH inhibitor iGP-1(100 μMm, Sigma Aldrich), without any detectable effects (data not shown).

Mitochondrial isolation: 25 to 30 *Drosophila* thoraces were freshly dissected and homogenized in isolation buffer (250 mM of sucrose, 5 mM of Trisma Base, 2 mM ethylene glycol-bis (β-aminoethyl ether)-N,N,N′,N′-tetraacetic acid (EGTA), pH 7.4) containing 1% (w/v) BSA, with a pellet pestle. Homogenates were filtered through a 1 mL syringe fitted with a piece of gauze (approx. 1 cm^2) and centrifuged at 310 x *g* for 3 min at 4 °C. The resulting supernatant was re-filtered and centrifuged at 9,000 x *g* for 10 min at 4 °C. The supernatant was then discarded, and the pellet containing mitochondria was washed and resuspended in isolation buffer, followed by centrifugation at 9000 x *g* for 10 min at 4 °C. Finally, the supernatant was discarded, and the pellet was washed and resuspended (4, 5). Protein content of mitochondrial preparations were assayed using the bicinchoninic acid method (3), with BSA as a standard.

ATP production: ATP production from isolated mitochondria were measured through the enzymatic coupled system described by Lark *et al*. 2016 (6). In this assay, hexokinase (HK) catalyzes the transformation of glucose into glucose-6-phosphate by using the ATP produced by the mitochondrion. Glucose-6-phosphate dehydrogenase (G6PDH) transfers electrons from glucose-6-phosphate to $NADP^+$, forming NADPH and 6-phospho-D-glucono-1,5-lactone. ATP production is thus stoichiometrically equivalent to NADPH production rate. Briefly, isolated mitochondria were incubated in respiration medium (120 mM KCl, 5 mM KH_2PO_4, 3 mM HEPES buffer, 1 mM $MgCl_2$, 0.2% BSA, pH 7.2) supplemented with glucose (20 mM) and HK (1.6 $kU.mL^{-1}$) as well as different substrates and inhibitors in order to evaluate the contribution of the different mitochondrial complexes of the ETS. Using a BioTek Synergy H1 microplate reader (Biotek®) set at 340 nm, we specifically measured the ATP production rate by: (i) CI+CII+mtG3PDH, in presence of pyruvate (10 mM), malate (1 mM), succinate (5 mM), G3P (7.5 mM) and ADP (5 mM); (ii) mtG3PDH in the presence of G3P (7.5 mM) and ADP (5 mM); and (iii) complex I in the presence of pyruvate (10 mM), malate (1 mM), and ADP (5 mM). Control conditions containing oligomycin (0.1 μM) and P1,P5-di(adenosine-5')pentaphosphate

(Ap5a, 0.5 mM) were performed in parallel to inhibit ATP synthase and adenylate kinase, respectively. Resulting mitochondrial ATP production is expressed as µmol. $min^{-1}.mg^{-1}$ of protein.

Mitochondrial O_2 consumption: Oxygen consumption rates were measured in isolated mitochondria from *D. melanogaster* thoraces using the Oxygraph-O2k system (7) (Oroboros Instruments, Innsbruck, Austria). Oxygraphs were first calibrated at 24 °C with respiration medium. To initiate measurements, 10 µL of isolated mitochondria samples (3.00 ± 0.57 mg protein. mL^{-1}) were added into the chamber, and once the signal was stable, different substrates and inhibitors were added. First, were added pyruvate (10 mM) and malate (2 mM) to stimulate oxygen consumption during LEAK respiration at the level of complex I (CI-LEAK). Then ADP (5 mM) was added to initiate oxidative phosphorylation by ATP synthase at the level of CI (CI-OXPHOS), followed by succinate (10 mM) and G3P (7.5 mM), to provide substrates to complex II (CI+CII-OXPHOS) and mtG3PDH (CI+CII+mt3GPDH-OXPHOS), respectively. Inhibitors were then added to sequentially inhibit ETS complexes and measure the residual oxygen consumption (ROX): first, rotenone (0.5 µM) inhibiting complex I; then malonate (5 mM) to inhibit complex II; and myxothiazol (0.5 µM), inhibiting complex III.

Analysis of O_2 consumption: The ROX was subtracted from all the substrate-specific oxygen consumption rates (OCR) to correct for oxygen consumed by non-mitochondrial reactions. O_2 consumption rates are expressed as pmol $O_2.s^{-1}.mg^{-1}$ of proteins. OCRs were used to calculate different ratios as previously described (8).

The OXPHOS coupling efficiency at the level of complex I was calculated as:

$$\text{CI coupling efficiency} = 1 - (\text{CI LEAK} / \text{CI OXPHOS}).$$

A ratio close to 1.0 indicates a tight coupling between the electron transport from complex I and the phosphorylation process.

We also estimated the contribution of G3P (G3P contribution ratio) to mitochondrial respiration, using the following respiration rates:

$$(\text{CI+CII+mtG3PDH-OXPHOS} - \text{CI+CII-OXPHOS})/\text{CI+CII+mtG3PDH-OXPHOS}$$

If this ratio is 1.0, the addition of G3P resulted in a 100% increase in oxygen consumption rate (i.e. doubled oxygen consumption).

Mitochondrial efficiency (ATP/O): The ATP/O ratio was obtain using the conditions where CI, CII and mtG3PDH are fueled. The ratio was calculated by dividing the ATP synthesis rate by half the corresponding oxygen consumption rate (OXPHOS) measured in the same mitochondrial sample, as oxygen consumption is expressed per O_2 rather than per atom of oxygen (O). While the concentrations of malate and succinate slightly differed between ATP production and oxygen consumption assays, they were stimulating the maximal rate in both cases.

H_2O_2 emission: H_2O_2 emission rates of isolated mitochondria were measured using Amplex®UltraRed reagent (Invitrogen™) and a BioTek Synergy H1 microplate reader (Biotek®) set at 24 °C with excitation/emission wavelengths set at 530/590 nm. Briefly, the superoxide ($O_2^{\cdot-}$) produced by mitochondria is catalyzed by superoxide dismutase (SOD) to form H_2O_2. This H_2O_2 is then reduced to form H_2O by horseradish peroxidase (HRP), oxidizing at the same time Amplex®UltraRed (530/590 nm) to form resorufin, which is then detected by fluorescence. Briefly, Amplex®UltraRed reagent (5 μM), HRP (1.5 $U.mL^{-1}$) and SOD (55 $U.mL^{-1}$) were added to respiration medium. Different combinations of mitochondrial substrates and inhibitors were added to measure site-specific reactive oxygen species (ROS) production (as H_2O_2 emission rates) during high membrane potential (i.e. without ADP and/or with oligomycin to inhibit ATP synthase). We specifically measured: (i) H_2O_2 emission rates with pyruvate (10 mM), malate (2 mM), succinate (5 mM) and G3P (7.5 mM): CI+CII+mtG3PDH; (ii) H_2O_2 emission rates with succinate (5 mM), G3P (7.5 mM) and GDP (1 mM): CII+mtG3PDH; (iii) H_2O_2 emission rates with succinate (5mM), rotenone (2mM), myxothiazol (4mM), antimycine A (2 mM) and oligomycine (1mM): CII; and (iv) H_2O_2 emission rates with G3P (7.5 mM), rotenone (1 μM), malonate (2 mM), myxothiazol (2 μM), antimycin A (5 μM) and oligomycin (5 nM): mtG3PDH (9). Standards with different H_2O_2 concentrations (0 to 5 μM) were used to express H_2O_2 emission rates as nmol $H_2O_2.min^{-1}.mg^{-1}$ of protein.

Statistical analysis: All statistical analyses were performed using R software (version 4.5.1, Free Software Foundation, Boston, MA, USA), with significance set at $p < 0.05$. Outliers were identified and removed using a Grubbs' test, while ensuring that each group retained a minimum of five samples.

To detect survival divergence between the different lines, a log-rank test was performed.

For enzymatic activity of mtG3PDH, a two-way ANOVA (including lines and protocols) was performed, followed by a post-hoc test of Tukey using the emmeans function to identify specific differences. Normality was verified using a Shapiro-Wilk test and homogeneity of variances was verified using Levene's test. Data were transformed to meet assumptions when required.

For climbing, mitochondrial data (ATP production, H_2O_2 emission, O_2 consumption, ratios), and enzymatic activity of cG3PDH, an unpaired bilateral student's t-test was performed to evaluate differences between lines. Normality and homoscedasticity were verified using Shapiro-Wilk test and Fisher's test, respectively. Data were transformed when necessary to meet assumptions. If no data transformation allowed the normality of data, a non-parametric Mann-Whitney test was performed. If homoscedasticity assumption was not met, a Welch t-test was performed. Significance levels are represented using asterisk ($p < 0.05$*; $p < 0.01$**; $p < 0.001$***).

Data availability

Data are available at Mendeley Data (DOI: 10.17632/7pdcjmc6x3.1).

References

1. R. P. J. Cormier, C. M. Champigny, C. J. Simard, P.-D. St-Coeur, N. Pichaud, Dynamic mitochondrial responses to a high-fat diet in Drosophila melanogaster. *Sci Rep* **9**, 4531 (2019).

2. A. L. Orr, *et al.*, Novel Inhibitors of Mitochondrial sn-Glycerol 3-phosphate Dehydrogenase. *PLoS ONE* **9**, e89938 (2014).

3. P. K. Smith, *et al.*, Measurement of protein using bicinchoninic acid. *Analytical Biochemistry* **150**, 76–85 (1985).

4. A. Léger, S. B. Cormier, A. Blanchard, H. A. Menail, N. Pichaud, Investigating the thermal sensitivity of key enzymes involved in the energetic metabolism of three insect species. *Journal of Experimental Biology* **227**, jeb247221 (2024).

5. D. Roussel, S. Janillon, L. Teulier, N. Pichaud, Succinate oxidation rescues mitochondrial ATP synthesis at high temperature in *Drosophila melanogaster*. *FEBS Letters* **597**, 2221–2229 (2023).

6. D. S. Lark, *et al.*, Direct real-time quantification of mitochondrial oxidative phosphorylation efficiency in permeabilized skeletal muscle myofibers. *American Journal of Physiology-Cell Physiology* **311**, C239–C245 (2016).

7. F. Hunter-Manseau, S. B. Cormier, R. Strang, N. Pichaud, Fasting as a precursor to high-fat diet enhances mitochondrial resilience in *Drosophila melanogaster*. *Insect Science* 1744-7917.13355 (2024). https://doi.org/10.1111/1744-7917.13355.

8. H. A. Menail, *et al.*, Flexible Thermal Sensitivity of Mitochondrial Oxygen Consumption and Substrate Oxidation in Flying Insect Species. *Front. Physiol.* **13**, 897174 (2022).

9. A. L. Orr, C. L. Quinlan, I. V. Perevoshchikova, M. D. Brand, A Refined Analysis of Superoxide Production by Mitochondrial sn-Glycerol 3-Phosphate Dehydrogenase. *Journal of Biological Chemistry* **287**, 42921–42935 (2012).
